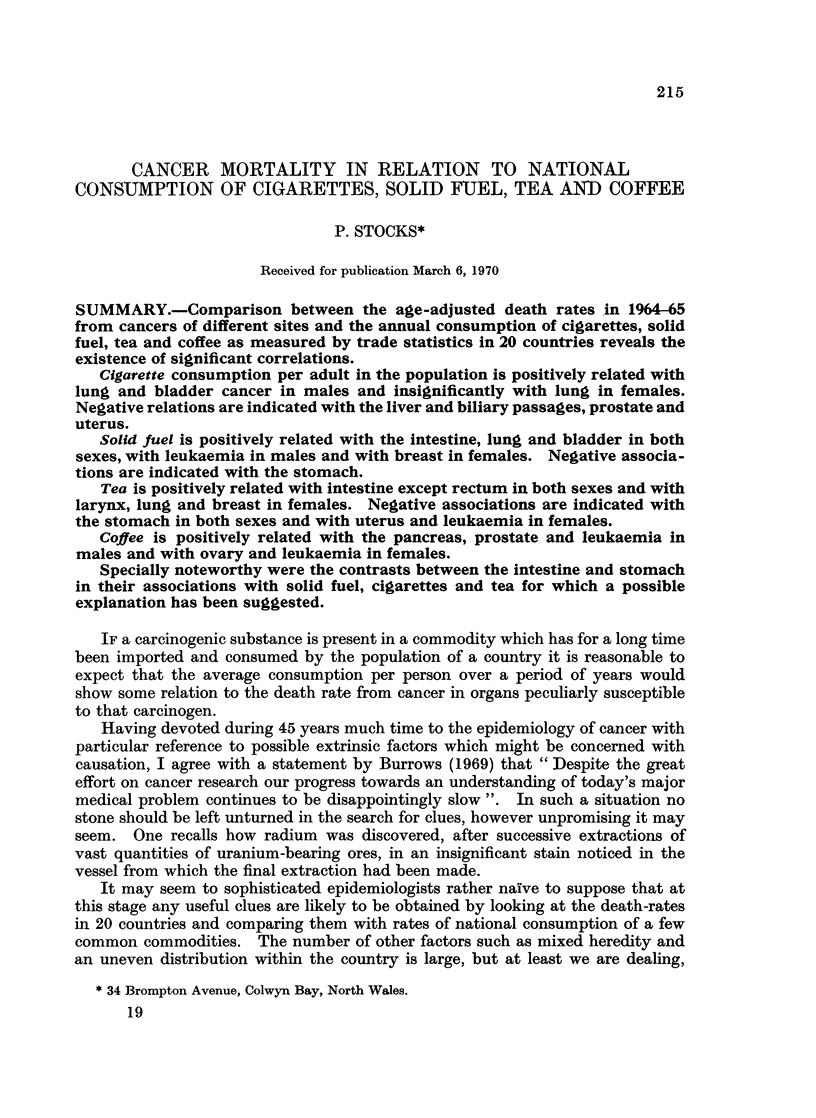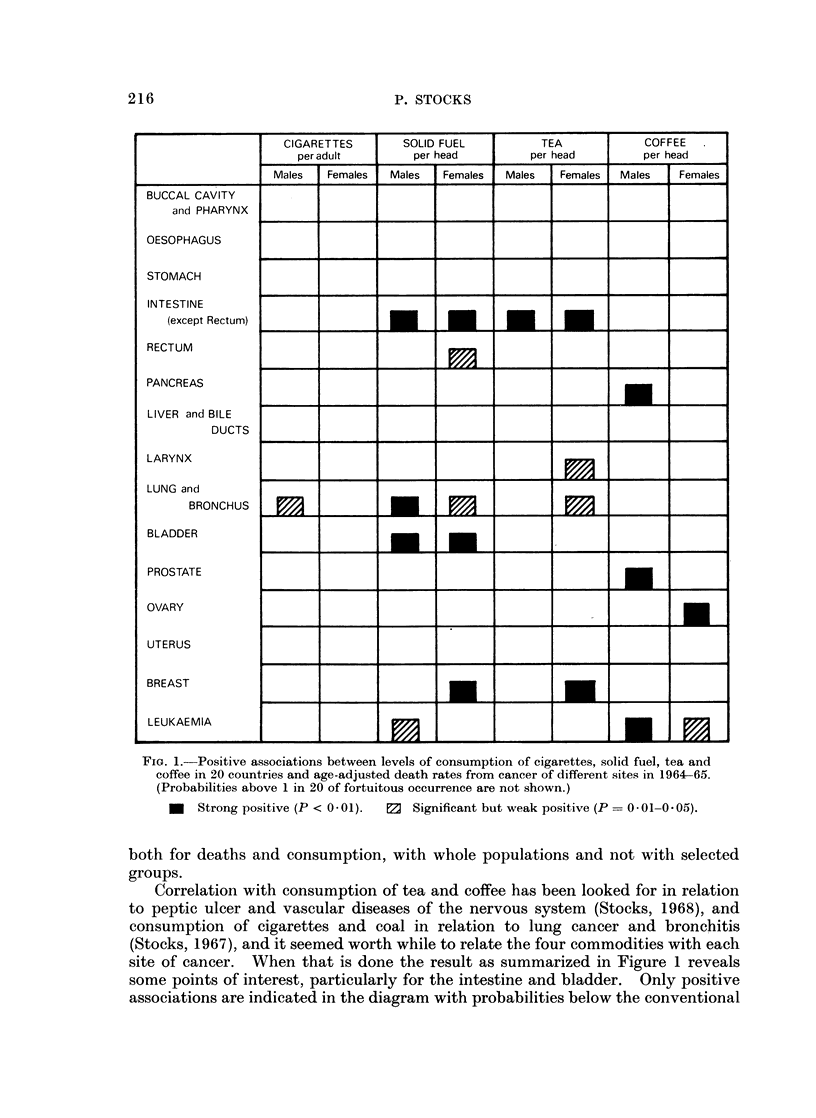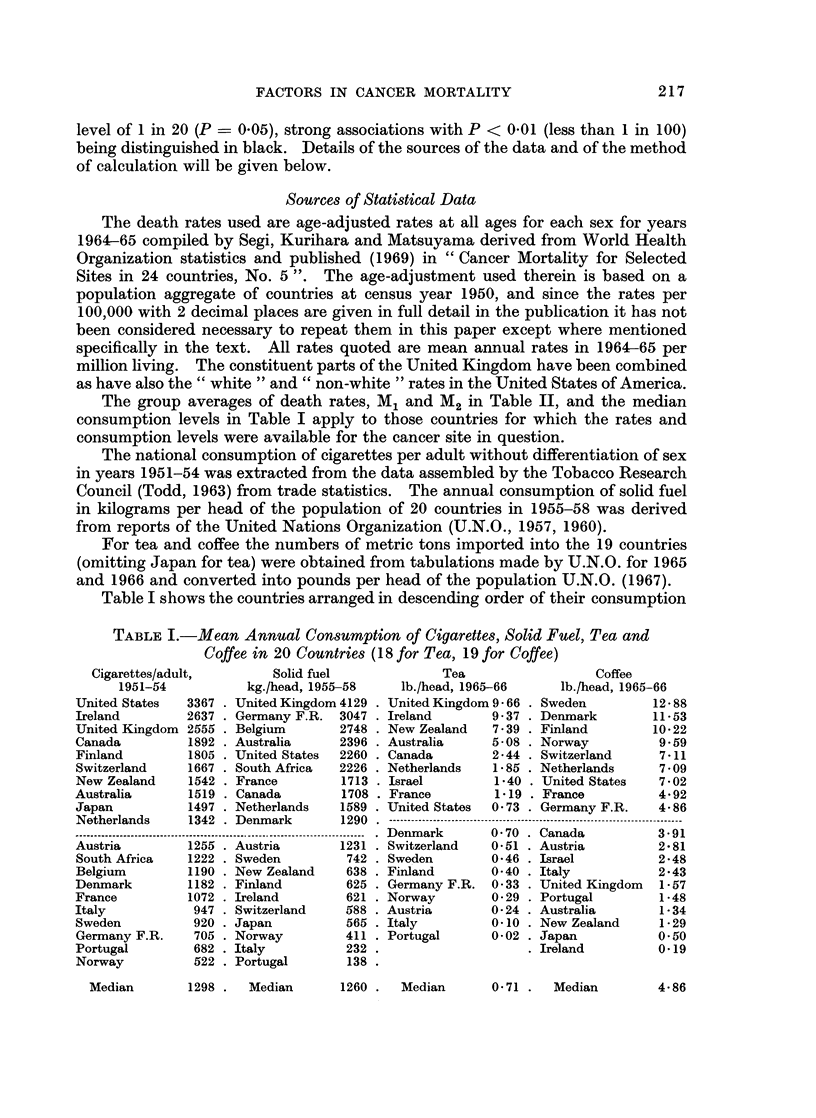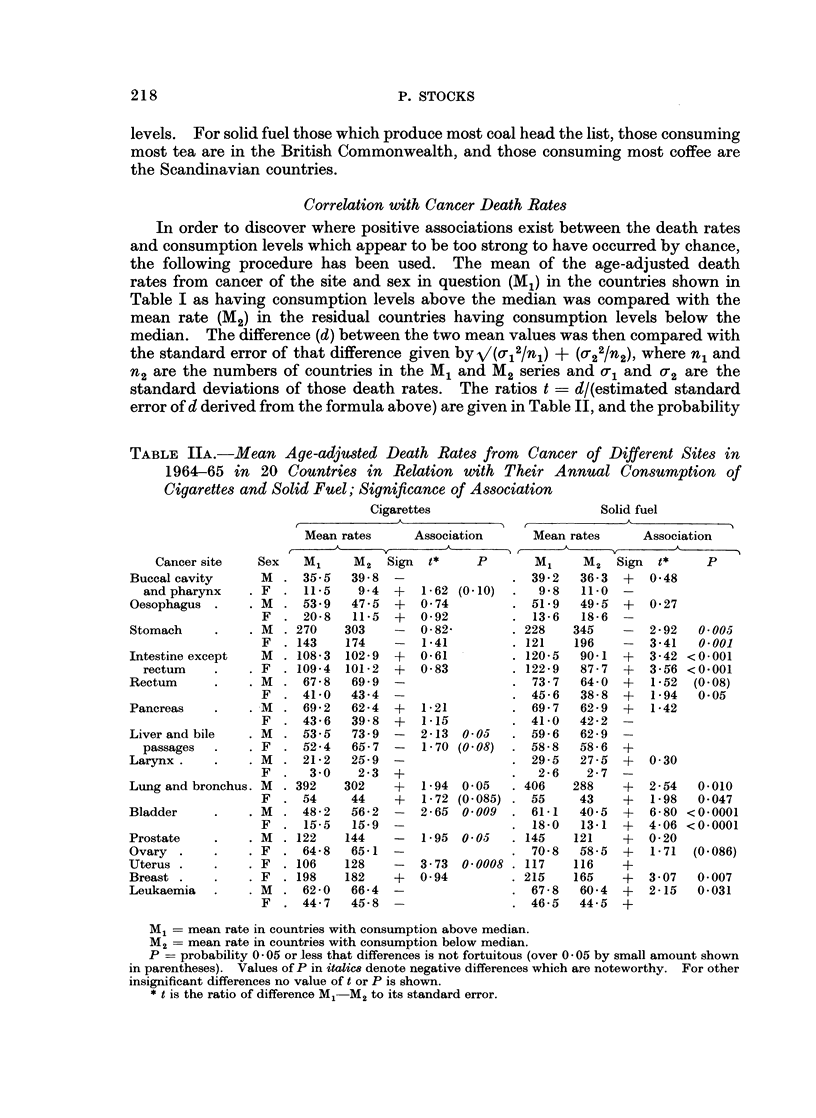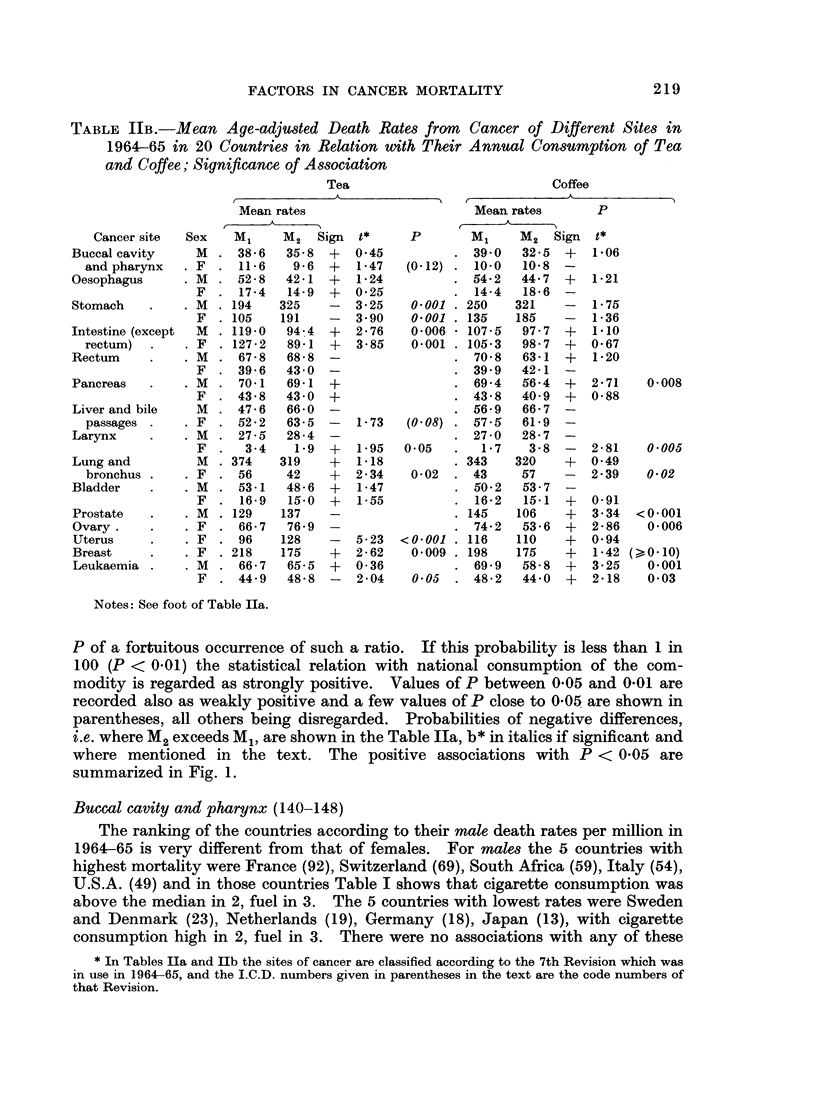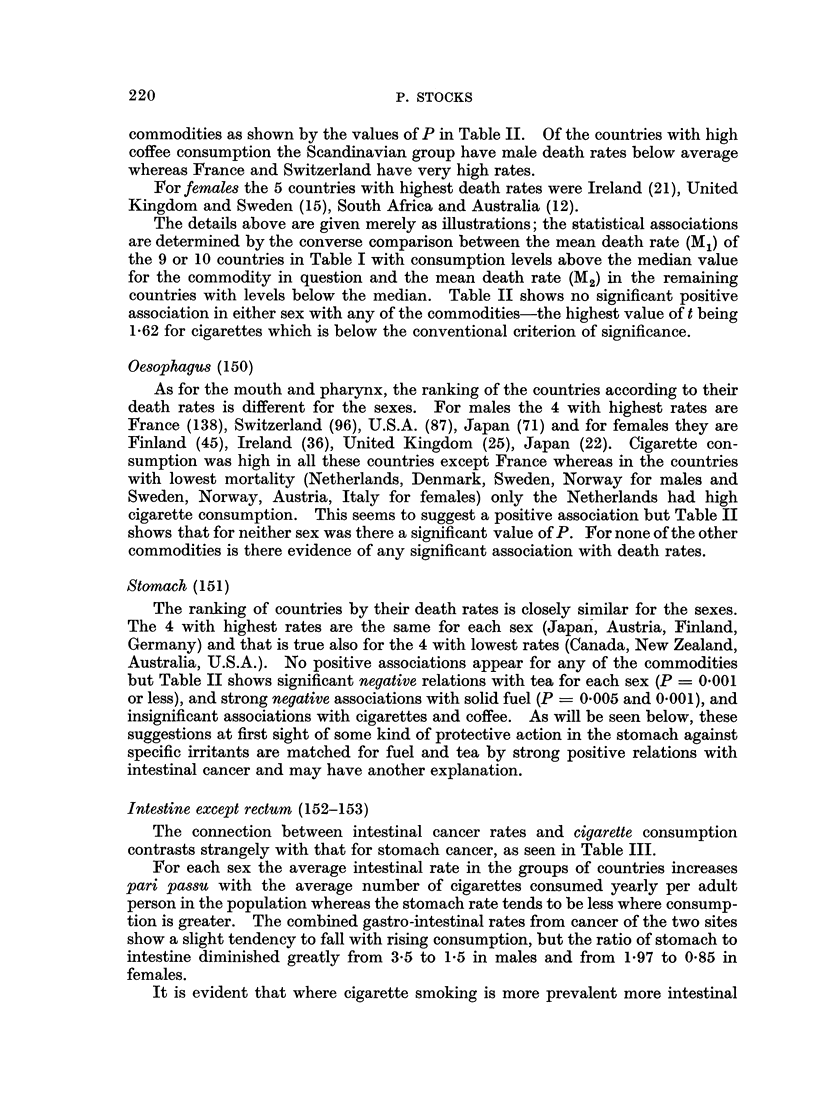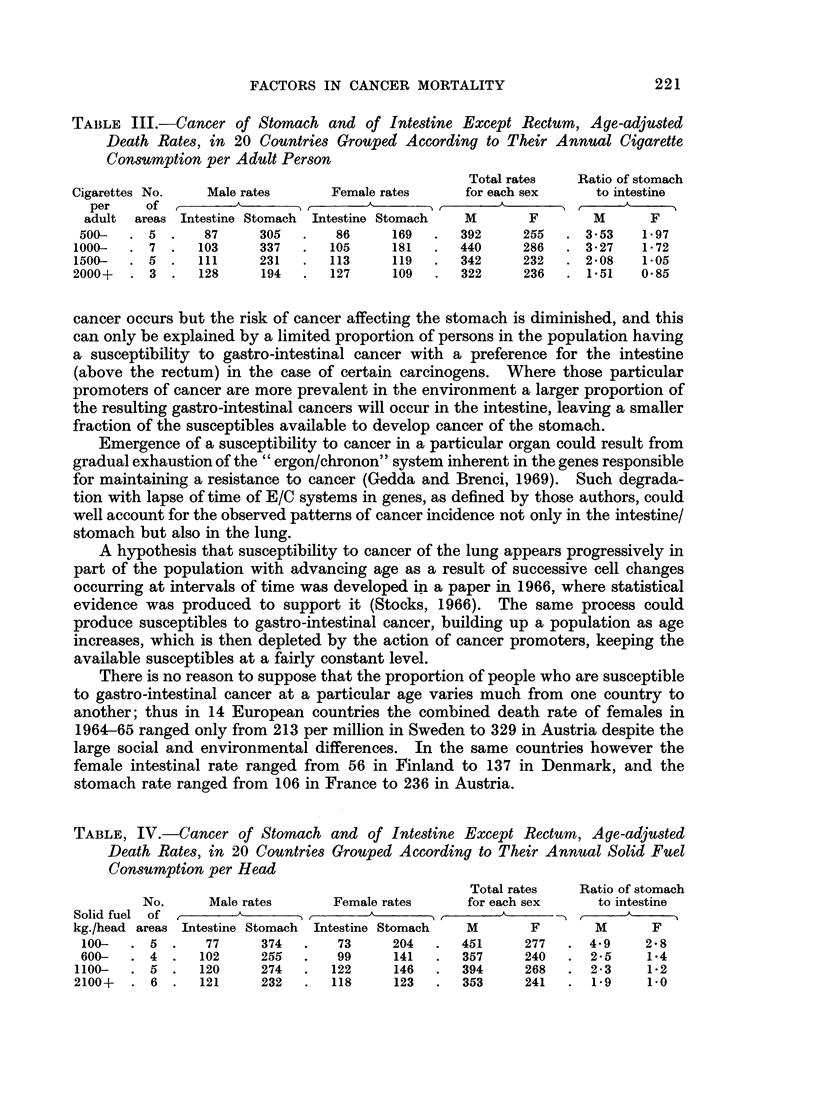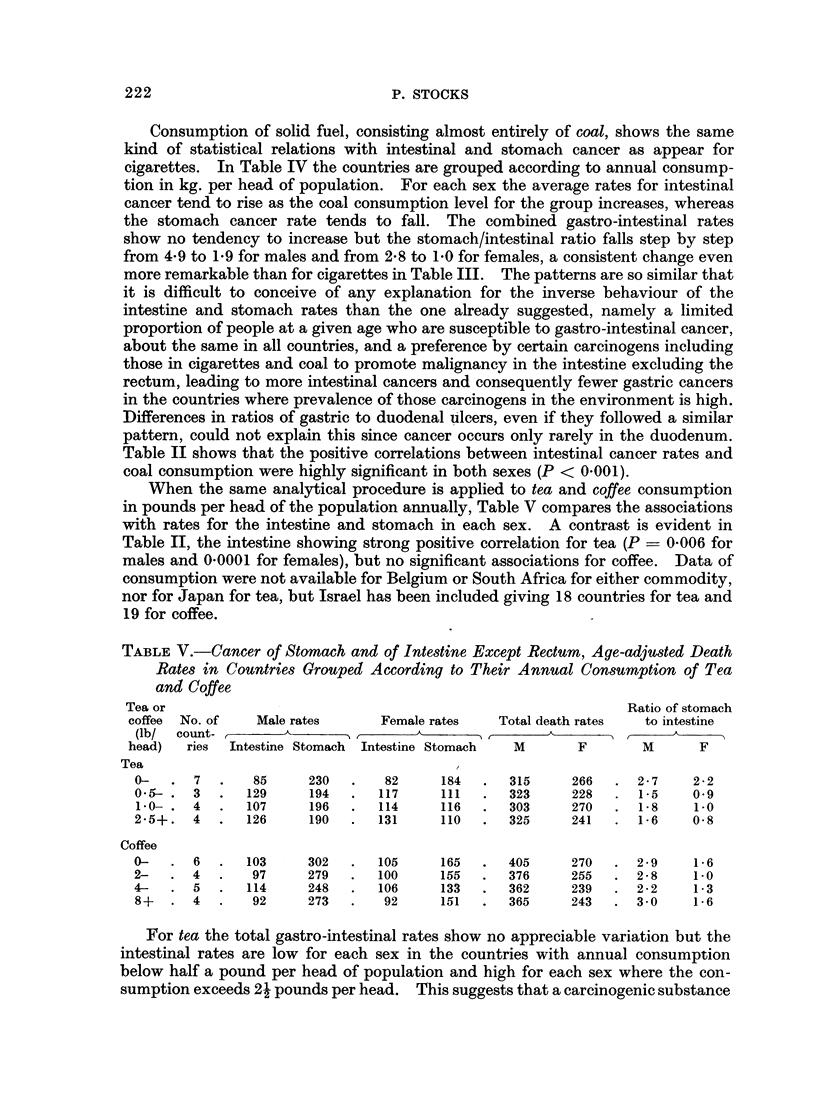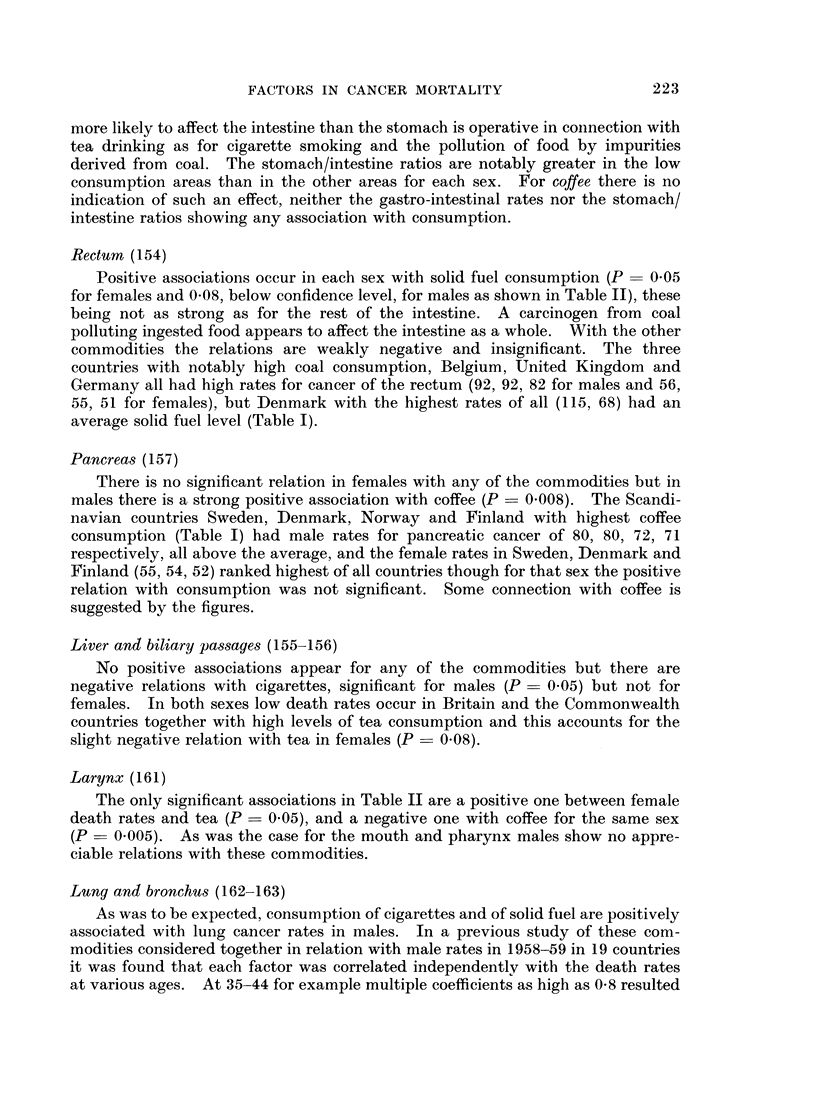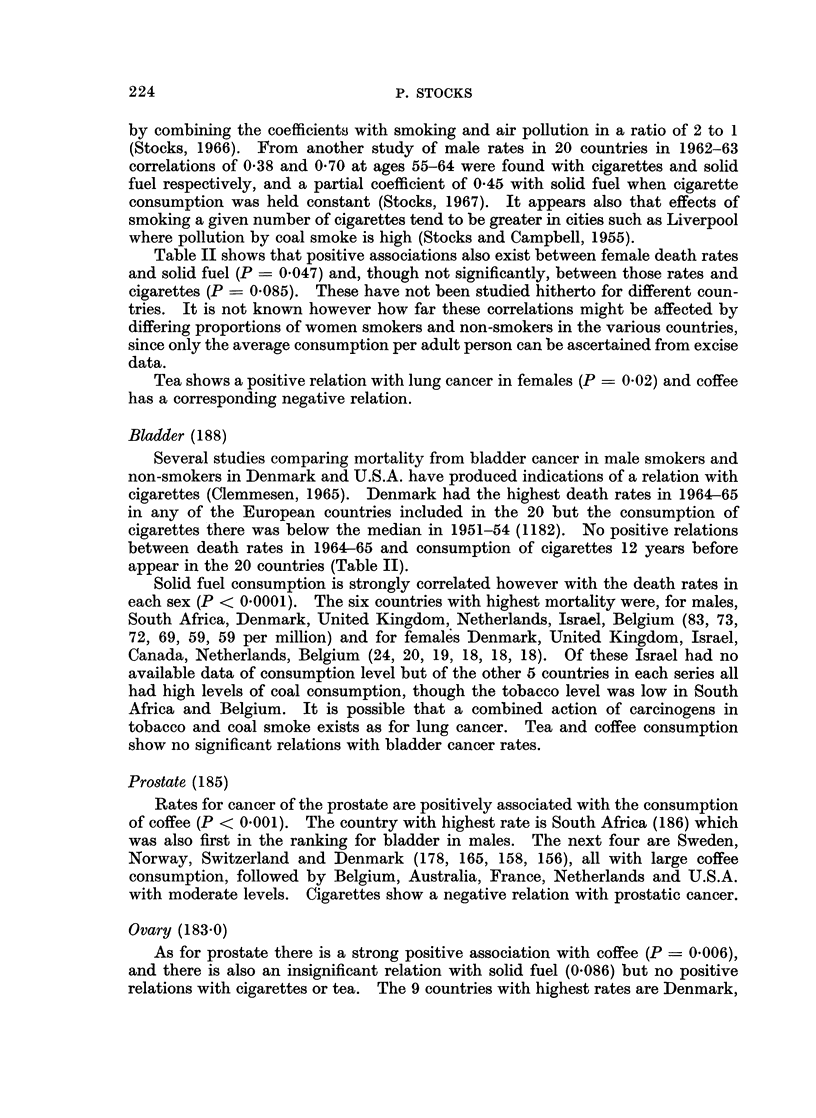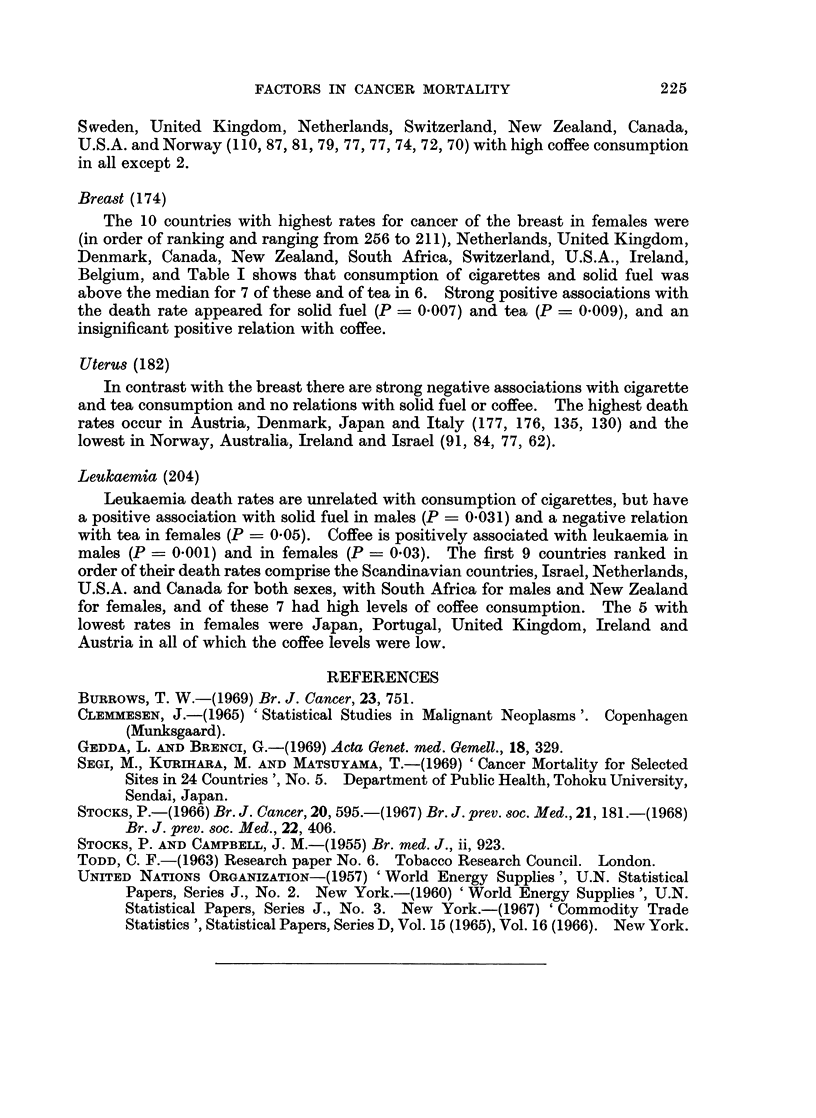# Cancer Mortality in Relation to National Consumption of Cigarettes, Solid Fuel, Tea and Coffee

**DOI:** 10.1038/bjc.1970.25

**Published:** 1970-06

**Authors:** P. Stocks

## Abstract

Comparison between the age-adjusted death rates in 1964-65 from cancers of different sites and the annual consumption of cigarettes, solid fuel, tea and coffee as measured by trade statistics in 20 countries reveals the existence of significant correlations.

*Cigarette* consumption per adult in the population is positively related with lung and bladder cancer in males and insignificantly with lung in females. Negative relations are indicated with the liver and biliary passages, prostate and uterus.

*Solid fuel* is positively related with the intestine, lung and bladder in both sexes, with leukaemia in males and with breast in females. Nagative associations are indicated with the stomach.

*Tea* is positively related with intestine except rectum in both sexes and with larynx, lung and breast in females. Negative associations are indicated with the stomach in both sexes and with uterus and leukaemia in females.

*Coffee* is positively related with the pancreas, prostate and leukaemia in males and with ovary and leukaemia in females.

Specially noteworthy were the contrasts between the intestine and stomach in their associations with solid fuel, cigarettes and tea for which a possible explanation has been suggested.


### CANCER MORTALITY IN RELATION TO NATIONAL CONSUMPTION OF CIGARETTES, SOLID FUEL, TEA AND COFFEE

P. STOCKS*

Received for publication March 6, 1970

**SUMMARY.—Comparison between the age-adjusted death rates in 1964–65 from cancers of different sites and the annual consumption of cigarettes, solid fuel, tea and coffee as measured by trade statistics in 20 countries reveals the existence of significant correlations.**

***Cigarette* consumption per adult in the population is positively related with lung and bladder cancer in males and insignificantly with lung in females. Negative relations are indicated with the liver and biliary passages, prostate and uterus.**

***Solid fuel* is positively related with the intestine, lung and bladder in both sexes, with leukaemia in males and with breast in females. Negative associations are indicated with the stomach.**

***Tea* is positively related with intestine except rectum in both sexes and with larynx, lung and breast in females. Negative associations are indicated with the stomach in both sexes and with uterus and leukaemia in females.**

***Coffee* is positively related with the pancreas, prostate and leukaemia in males and with ovary and leukaemia in females.**

**Specially noteworthy were the contrasts between the intestine and stomach in their associations with solid fuel, cigarettes and tea for which a possible explanation has been suggested.**

IF a carcinogenic substance is present in a commodity which has for a long time been imported and consumed by the population of a country it is reasonable to expect that the average consumption per person over a period of years would show some relation to the death rate from cancer in organs peculiarly susceptible to that carcinogen.

Having devoted during 45 years much time to the epidemiology of cancer with particular reference to possible extrinsic factors which might be concerned with causation, I agree with a statement by Burrows (1969) that " Despite the great effort on cancer research our progress towards an understanding of today's major medical problem continues to be disappointingly slow ". In such a situation no stone should be left unturned in the search for clues, however unpromising it may seem. One recalls how radium was discovered, after successive extractions of vast quantities of uranium-bearing ores, in an insignificant stain noticed in the vessel from which the final extraction had been made.

It may seem to sophisticated epidemiologists rather naïve to suppose that at this stage any useful clues are likely to be obtained by looking at the death-rates in 20 countries and comparing them with rates of national consumption of a few common commodities. The number of other factors such as mixed heredity and an uneven distribution within the country is large, but at least we are dealing,

* 34 Brompton Avenue, Colwyn Bay, North Wales.

| | CIGARETTES per adult | | SOLID FUEL per head | | TEA per head | | COFFEE per head | |
|---|---|---|---|---|---|---|---|---|
| | Males | Females | Males | Females | Males | Females | Males | Females |
| BUCCAL CAVITY and PHARYNX | | | | | | | | |
| OESOPHAGUS | | | | | | | | |
| STOMACH | | | | | | | | |
| INTESTINE (except Rectum) | | | ■ | ■ | ■ | ■ | | |
| RECTUM | | | | ▨ | | | | |
| PANCREAS | | | | | | | ■ | |
| LIVER and BILE DUCTS | | | | | | | | |
| LARYNX | | | | | | ▨ | | |
| LUNG and BRONCHUS | ▨ | | ■ | ▨ | | ▨ | | |
| BLADDER | | | ■ | ■ | | | | |
| PROSTATE | | | | | | | ■ | |
| OVARY | | | | | | | | ■ |
| UTERUS | | | | | | | | |
| BREAST | | | | ■ | | ■ | | |
| LEUKAEMIA | | | ▨ | | | | ■ | ▨ |

Fig. 1.—Positive associations between levels of consumption of cigarettes, solid fuel, tea and coffee in 20 countries and age-adjusted death rates from cancer of different sites in 1964–65. (Probabilities above 1 in 20 of fortuitous occurrence are not shown.)

■ Strong positive ($P < 0\cdot01$). ▨ Significant but weak positive ($P = 0\cdot01$–$0\cdot05$).

both for deaths and consumption, with whole populations and not with selected groups.

Correlation with consumption of tea and coffee has been looked for in relation to peptic ulcer and vascular diseases of the nervous system (Stocks, 1968), and consumption of cigarettes and coal in relation to lung cancer and bronchitis (Stocks, 1967), and it seemed worth while to relate the four commodities with each site of cancer. When that is done the result as summarized in Figure 1 reveals some points of interest, particularly for the intestine and bladder. Only positive associations are indicated in the diagram with probabilities below the conventional level of 1 in 20 ($P = 0{\cdot}05$), strong associations with $P < 0{\cdot}01$ (less than 1 in 100) being distinguished in black. Details of the sources of the data and of the method of calculation will be given below.

*Sources of Statistical Data*

The death rates used are age-adjusted rates at all ages for each sex for years 1964–65 compiled by Segi, Kurihara and Matsuyama derived from World Health Organization statistics and published (1969) in " Cancer Mortality for Selected Sites in 24 countries, No. 5 ". The age-adjustment used therein is based on a population aggregate of countries at census year 1950, and since the rates per 100,000 with 2 decimal places are given in full detail in the publication it has not been considered necessary to repeat them in this paper except where mentioned specifically in the text. All rates quoted are mean annual rates in 1964–65 per million living. The constituent parts of the United Kingdom have been combined as have also the " white " and " non-white " rates in the United States of America.

The group averages of death rates, $M_1$ and $M_2$ in Table II, and the median consumption levels in Table I apply to those countries for which the rates and consumption levels were available for the cancer site in question.

The national consumption of cigarettes per adult without differentiation of sex in years 1951–54 was extracted from the data assembled by the Tobacco Research Council (Todd, 1963) from trade statistics. The annual consumption of solid fuel in kilograms per head of the population of 20 countries in 1955–58 was derived from reports of the United Nations Organization (U.N.O., 1957, 1960).

For tea and coffee the numbers of metric tons imported into the 19 countries (omitting Japan for tea) were obtained from tabulations made by U.N.O. for 1965 and 1966 and converted into pounds per head of the population U.N.O. (1967).

Table I shows the countries arranged in descending order of their consumption

TABLE I.—*Mean Annual Consumption of Cigarettes, Solid Fuel, Tea and Coffee in 20 Countries* (18 *for Tea,* 19 *for Coffee*)

| Cigarettes/adult, 1951–54 | | Solid fuel kg./head, 1955–58 | | Tea lb./head, 1965–66 | | Coffee lb./head, 1965–66 | |
|---|---|---|---|---|---|---|---|
| United States | 3367 | United Kingdom | 4129 | United Kingdom | 9·66 | Sweden | 12·88 |
| Ireland | 2637 | Germany F.R. | 3047 | Ireland | 9·37 | Denmark | 11·53 |
| United Kingdom | 2555 | Belgium | 2748 | New Zealand | 7·39 | Finland | 10·22 |
| Canada | 1892 | Australia | 2396 | Australia | 5·08 | Norway | 9·59 |
| Finland | 1805 | United States | 2260 | Canada | 2·44 | Switzerland | 7·11 |
| Switzerland | 1667 | South Africa | 2226 | Netherlands | 1·85 | Netherlands | 7·09 |
| New Zealand | 1542 | France | 1713 | Israel | 1·40 | United States | 7·02 |
| Australia | 1519 | Canada | 1708 | France | 1·19 | France | 4·92 |
| Japan | 1497 | Netherlands | 1589 | United States | 0·73 | Germany F.R. | 4·86 |
| Netherlands | 1342 | Denmark | 1290 | ----- | ----- | ----- | ----- |
| ----- | ----- | ----- | ----- | Denmark | 0·70 | Canada | 3·91 |
| Austria | 1255 | Austria | 1231 | Switzerland | 0·51 | Austria | 2·81 |
| South Africa | 1222 | Sweden | 742 | Sweden | 0·46 | Israel | 2·48 |
| Belgium | 1190 | New Zealand | 638 | Finland | 0·40 | Italy | 2·43 |
| Denmark | 1182 | Finland | 625 | Germany F.R. | 0·33 | United Kingdom | 1·57 |
| France | 1072 | Ireland | 621 | Norway | 0·29 | Portugal | 1·48 |
| Italy | 947 | Switzerland | 588 | Austria | 0·24 | Australia | 1·34 |
| Sweden | 920 | Japan | 565 | Italy | 0·10 | New Zealand | 1·29 |
| Germany F.R. | 705 | Norway | 411 | Portugal | 0·02 | Japan | 0·50 |
| Portugal | 682 | Italy | 232 | | | Ireland | 0·19 |
| Norway | 522 | Portugal | 138 | | | | |
| Median | 1298 | Median | 1260 | Median | 0·71 | Median | 4·86 |

levels. For solid fuel those which produce most coal head the list, those consuming most tea are in the British Commonwealth, and those consuming most coffee are the Scandinavian countries.

*Correlation with Cancer Death Rates*

In order to discover where positive associations exist between the death rates and consumption levels which appear to be too strong to have occurred by chance, the following procedure has been used. The mean of the age-adjusted death rates from cancer of the site and sex in question ($M_1$) in the countries shown in Table I as having consumption levels above the median was compared with the mean rate ($M_2$) in the residual countries having consumption levels below the median. The difference ($d$) between the two mean values was then compared with the standard error of that difference given by $\sqrt{(\sigma_1^2/n_1) + (\sigma_2^2/n_2)}$, where $n_1$ and $n_2$ are the numbers of countries in the $M_1$ and $M_2$ series and $\sigma_1$ and $\sigma_2$ are the standard deviations of those death rates. The ratios $t = d/$(estimated standard error of $d$ derived from the formula above) are given in Table II, and the probability

TABLE IIA.—*Mean Age-adjusted Death Rates from Cancer of Different Sites in 1964–65 in 20 Countries in Relation with Their Annual Consumption of Cigarettes and Solid Fuel; Significance of Association*

| | | Cigarettes | | | | | Solid fuel | | | | |
|---|---|---|---|---|---|---|---|---|---|---|---|
| | | Mean rates | | Association | | | Mean rates | | Association | | |
| Cancer site | Sex | $M_1$ | $M_2$ | Sign | $t$* | $P$ | $M_1$ | $M_2$ | Sign | $t$* | $P$ |
| Buccal cavity | M | 35·5 | 39·8 | – | | | 39·2 | 36·3 | + | 0·48 | |
| and pharynx | F | 11·5 | 9·4 | + | 1·62 | (0·10) | 9·8 | 11·0 | – | | |
| Oesophagus | M | 53·9 | 47·5 | + | 0·74 | | 51·9 | 49·5 | + | 0·27 | |
| | F | 20·8 | 11·5 | + | 0·92 | | 13·6 | 18·6 | – | | |
| Stomach | M | 270 | 303 | – | 0·82· | | 228 | 345 | – | 2·92 | *0·005* |
| | F | 143 | 174 | – | 1·41 | | 121 | 196 | – | 3·41 | *0·001* |
| Intestine except | M | 108·3 | 102·9 | + | 0·61 | | 120·5 | 90·1 | + | 3·42 | < 0·001 |
| rectum | F | 109·4 | 101·2 | + | 0·83 | | 122·9 | 87·7 | + | 3·56 | < 0·001 |
| Rectum | M | 67·8 | 69·9 | – | | | 73·7 | 64·0 | + | 1·52 | (0·08) |
| | F | 41·0 | 43·4 | – | | | 45·6 | 38·8 | + | 1·94 | 0·05 |
| Pancreas | M | 69·2 | 62·4 | + | 1·21 | | 69·7 | 62·9 | + | 1·42 | |
| | F | 43·6 | 39·8 | + | 1·15 | | 41·0 | 42·2 | – | | |
| Liver and bile | M | 53·5 | 73·9 | – | 2·13 | *0·05* | 59·6 | 62·9 | – | | |
| passages | F | 52·4 | 65·7 | – | 1·70 | *(0·08)* | 58·8 | 58·6 | + | | |
| Larynx | M | 21·2 | 25·9 | – | | | 29·5 | 27·5 | + | 0·30 | |
| | F | 3·0 | 2·3 | + | | | 2·6 | 2·7 | – | | |
| Lung and bronchus | M | 392 | 302 | + | 1·94 | 0·05 | 406 | 288 | + | 2·54 | 0·010 |
| | F | 54 | 44 | + | 1·72 | (0·085) | 55 | 43 | + | 1·98 | 0·047 |
| Bladder | M | 48·2 | 56·2 | – | 2·65 | *0·009* | 61·1 | 40·5 | + | 6·80 | < 0·0001 |
| | F | 15·5 | 15·9 | – | | | 18·0 | 13·1 | + | 4·06 | < 0·0001 |
| Prostate | M | 122 | 144 | – | 1·95 | *0·05* | 145 | 121 | + | 0·20 | |
| Ovary | F | 64·8 | 65·1 | – | | | 70·8 | 58·5 | + | 1·71 | (0·086) |
| Uterus | F | 106 | 128 | – | 3·73 | *0·0008* | 117 | 116 | + | | |
| Breast | F | 198 | 182 | + | 0·94 | | 215 | 165 | + | 3·07 | 0·007 |
| Leukaemia | M | 62·0 | 66·4 | – | | | 67·8 | 60·4 | + | 2·15 | 0·031 |
| | F | 44·7 | 45·8 | – | | | 46·5 | 44·5 | + | | |

$M_1$ = mean rate in countries with consumption above median.
$M_2$ = mean rate in countries with consumption below median.
$P$ = probability 0·05 or less that differences is not fortuitous (over 0·05 by small amount shown in parentheses). Values of $P$ in *italics* denote negative differences which are noteworthy. For other insignificant differences no value of $t$ or $P$ is shown.
* $t$ is the ratio of difference $M_1$—$M_2$ to its standard error.

TABLE IIb.—*Mean Age-adjusted Death Rates from Cancer of Different Sites in 1964–65 in 20 Countries in Relation with Their Annual Consumption of Tea and Coffee; Significance of Association*

| | | Tea | | | | | Coffee | | | | |
|---|---|---|---|---|---|---|---|---|---|---|---|
| | | Mean rates | | | | | Mean rates | | | P | |
| Cancer site | Sex | $M_1$ | $M_2$ | Sign | t* | P | $M_1$ | $M_2$ | Sign | t* | |
| Buccal cavity | M | 38·6 | 35·8 | + | 0·45 | | 39·0 | 32·5 | + | 1·06 | |
| and pharynx | F | 11·6 | 9·6 | + | 1·47 | (0·12) | 10·0 | 10·8 | − | | |
| Oesophagus | M | 52·8 | 42·1 | + | 1·24 | | 54·2 | 44·7 | + | 1·21 | |
| | F | 17·4 | 14·9 | + | 0·25 | | 14·4 | 18·6 | − | | |
| Stomach | M | 194 | 325 | − | 3·25 | *0·001* | 250 | 321 | − | 1·75 | |
| | F | 105 | 191 | − | 3·90 | *0·001* | 135 | 185 | − | 1·36 | |
| Intestine (except | M | 119·0 | 94·4 | + | 2·76 | 0·006 | 107·5 | 97·7 | + | 1·10 | |
| rectum) | F | 127·2 | 89·1 | + | 3·85 | 0·001 | 105·3 | 98·7 | + | 0·67 | |
| Rectum | M | 67·8 | 68·8 | − | | | 70·8 | 63·1 | + | 1·20 | |
| | F | 39·6 | 43·0 | − | | | 39·9 | 42·1 | − | | |
| Pancreas | M | 70·1 | 69·1 | + | | | 69·4 | 56·4 | + | 2·71 | 0·008 |
| | F | 43·8 | 43·0 | + | | | 43·8 | 40·9 | + | 0·88 | |
| Liver and bile | M | 47·6 | 66·0 | − | | | 56·9 | 66·7 | − | | |
| passages | F | 52·2 | 63·5 | − | 1·73 | *(0·08)* | 57·5 | 61·9 | − | | |
| Larynx | M | 27·5 | 28·4 | − | | | 27·0 | 28·7 | − | | |
| | F | 3·4 | 1·9 | + | 1·95 | 0·05 | 1·7 | 3·8 | − | 2·81 | *0·005* |
| Lung and | M | 374 | 319 | + | 1·18 | | 343 | 320 | + | 0·49 | |
| bronchus | F | 56 | 42 | + | 2·34 | 0·02 | 43 | 57 | − | 2·39 | *0·02* |
| Bladder | M | 53·1 | 48·6 | + | 1·47 | | 50·2 | 53·7 | − | | |
| | F | 16·9 | 15·0 | + | 1·55 | | 16·2 | 15·1 | + | 0·91 | |
| Prostate | M | 129 | 137 | − | | | 145 | 106 | + | 3·34 | $<$0·001 |
| Ovary | F | 66·7 | 76·9 | − | | | 74·2 | 53·6 | + | 2·86 | 0·006 |
| Uterus | F | 96 | 128 | − | 5·23 | *$<$0·001* | 116 | 110 | + | 0·94 | |
| Breast | F | 218 | 175 | + | 2·62 | 0·009 | 198 | 175 | + | 1·42 | ($\geq$0·10) |
| Leukaemia | M | 66·7 | 65·5 | + | 0·36 | | 69·9 | 58·8 | + | 3·25 | 0·001 |
| | F | 44·9 | 48·8 | − | 2·04 | *0·05* | 48·2 | 44·0 | + | 2·18 | 0·03 |

Notes: See foot of Table IIa.

*P* of a fortuitous occurrence of such a ratio. If this probability is less than 1 in 100 ($P < 0·01$) the statistical relation with national consumption of the commodity is regarded as strongly positive. Values of *P* between 0·05 and 0·01 are recorded also as weakly positive and a few values of *P* close to 0·05 are shown in parentheses, all others being disregarded. Probabilities of negative differences, *i.e.* where $M_2$ exceeds $M_1$, are shown in the Table IIa, b* in italics if significant and where mentioned in the text. The positive associations with $P < 0·05$ are summarized in Fig. 1.

*Buccal cavity and pharynx* (140–148)

The ranking of the countries according to their *male* death rates per million in 1964–65 is very different from that of females. For *males* the 5 countries with highest mortality were France (92), Switzerland (69), South Africa (59), Italy (54), U.S.A. (49) and in those countries Table I shows that cigarette consumption was above the median in 2, fuel in 3. The 5 countries with lowest rates were Sweden and Denmark (23), Netherlands (19), Germany (18), Japan (13), with cigarette consumption high in 2, fuel in 3. There were no associations with any of these

* In Tables IIa and IIb the sites of cancer are classified according to the 7th Revision which was in use in 1964–65, and the I.C.D. numbers given in parentheses in the text are the code numbers of that Revision.

commodities as shown by the values of $P$ in Table II. Of the countries with high coffee consumption the Scandinavian group have male death rates below average whereas France and Switzerland have very high rates.

For *females* the 5 countries with highest death rates were Ireland (21), United Kingdom and Sweden (15), South Africa and Australia (12).

The details above are given merely as illustrations; the statistical associations are determined by the converse comparison between the mean death rate ($M_1$) of the 9 or 10 countries in Table I with consumption levels above the median value for the commodity in question and the mean death rate ($M_2$) in the remaining countries with levels below the median. Table II shows no significant positive association in either sex with any of the commodities—the highest value of $t$ being 1·62 for cigarettes which is below the conventional criterion of significance.

*Oesophagus* (150)

As for the mouth and pharynx, the ranking of the countries according to their death rates is different for the sexes. For males the 4 with highest rates are France (138), Switzerland (96), U.S.A. (87), Japan (71) and for females they are Finland (45), Ireland (36), United Kingdom (25), Japan (22). Cigarette consumption was high in all these countries except France whereas in the countries with lowest mortality (Netherlands, Denmark, Sweden, Norway for males and Sweden, Norway, Austria, Italy for females) only the Netherlands had high cigarette consumption. This seems to suggest a positive association but Table II shows that for neither sex was there a significant value of $P$. For none of the other commodities is there evidence of any significant association with death rates.

*Stomach* (151)

The ranking of countries by their death rates is closely similar for the sexes. The 4 with highest rates are the same for each sex (Japan, Austria, Finland, Germany) and that is true also for the 4 with lowest rates (Canada, New Zealand, Australia, U.S.A.). No positive associations appear for any of the commodities but Table II shows significant *negative* relations with tea for each sex ($P = 0·001$ or less), and strong *negative* associations with solid fuel ($P = 0·005$ and 0·001), and insignificant associations with cigarettes and coffee. As will be seen below, these suggestions at first sight of some kind of protective action in the stomach against specific irritants are matched for fuel and tea by strong positive relations with intestinal cancer and may have another explanation.

*Intestine except rectum* (152–153)

The connection between intestinal cancer rates and *cigarette* consumption contrasts strangely with that for stomach cancer, as seen in Table III.

For each sex the average intestinal rate in the groups of countries increases *pari passu* with the average number of cigarettes consumed yearly per adult person in the population whereas the stomach rate tends to be less where consumption is greater. The combined gastro-intestinal rates from cancer of the two sites show a slight tendency to fall with rising consumption, but the ratio of stomach to intestine diminished greatly from 3·5 to 1·5 in males and from 1·97 to 0·85 in females.

It is evident that where cigarette smoking is more prevalent more intestinal

TABLE III.—*Cancer of Stomach and of Intestine Except Rectum, Age-adjusted Death Rates, in 20 Countries Grouped According to Their Annual Cigarette Consumption per Adult Person*

| Cigarettes per adult | No. of areas | Male rates | | Female rates | | Total rates for each sex | | Ratio of stomach to intestine | |
|---|---|---|---|---|---|---|---|---|---|
| | | Intestine | Stomach | Intestine | Stomach | M | F | M | F |
| 500– | 5 | 87 | 305 | 86 | 169 | 392 | 255 | 3·53 | 1·97 |
| 1000– | 7 | 103 | 337 | 105 | 181 | 440 | 286 | 3·27 | 1·72 |
| 1500– | 5 | 111 | 231 | 113 | 119 | 342 | 232 | 2·08 | 1·05 |
| 2000+ | 3 | 128 | 194 | 127 | 109 | 322 | 236 | 1·51 | 0·85 |

cancer occurs but the risk of cancer affecting the stomach is diminished, and this can only be explained by a limited proportion of persons in the population having a susceptibility to gastro-intestinal cancer with a preference for the intestine (above the rectum) in the case of certain carcinogens. Where those particular promoters of cancer are more prevalent in the environment a larger proportion of the resulting gastro-intestinal cancers will occur in the intestine, leaving a smaller fraction of the susceptibles available to develop cancer of the stomach.

Emergence of a susceptibility to cancer in a particular organ could result from gradual exhaustion of the "ergon/chronon" system inherent in the genes responsible for maintaining a resistance to cancer (Gedda and Brenci, 1969). Such degradation with lapse of time of E/C systems in genes, as defined by those authors, could well account for the observed patterns of cancer incidence not only in the intestine/stomach but also in the lung.

A hypothesis that susceptibility to cancer of the lung appears progressively in part of the population with advancing age as a result of successive cell changes occurring at intervals of time was developed in a paper in 1966, where statistical evidence was produced to support it (Stocks, 1966). The same process could produce susceptibles to gastro-intestinal cancer, building up a population as age increases, which is then depleted by the action of cancer promoters, keeping the available susceptibles at a fairly constant level.

There is no reason to suppose that the proportion of people who are susceptible to gastro-intestinal cancer at a particular age varies much from one country to another; thus in 14 European countries the combined death rate of females in 1964–65 ranged only from 213 per million in Sweden to 329 in Austria despite the large social and environmental differences. In the same countries however the female intestinal rate ranged from 56 in Finland to 137 in Denmark, and the stomach rate ranged from 106 in France to 236 in Austria.

TABLE, IV.—*Cancer of Stomach and of Intestine Except Rectum, Age-adjusted Death Rates, in 20 Countries Grouped According to Their Annual Solid Fuel Consumption per Head*

| Solid fuel kg./head | No. of areas | Male rates | | Female rates | | Total rates for each sex | | Ratio of stomach to intestine | |
|---|---|---|---|---|---|---|---|---|---|
| | | Intestine | Stomach | Intestine | Stomach | M | F | M | F |
| 100– | 5 | 77 | 374 | 73 | 204 | 451 | 277 | 4·9 | 2·8 |
| 600– | 4 | 102 | 255 | 99 | 141 | 357 | 240 | 2·5 | 1·4 |
| 1100– | 5 | 120 | 274 | 122 | 146 | 394 | 268 | 2·3 | 1·2 |
| 2100+ | 6 | 121 | 232 | 118 | 123 | 353 | 241 | 1·9 | 1·0 |

Consumption of solid fuel, consisting almost entirely of *coal*, shows the same kind of statistical relations with intestinal and stomach cancer as appear for cigarettes. In Table IV the countries are grouped according to annual consumption in kg. per head of population. For each sex the average rates for intestinal cancer tend to rise as the coal consumption level for the group increases, whereas the stomach cancer rate tends to fall. The combined gastro-intestinal rates show no tendency to increase but the stomach/intestinal ratio falls step by step from 4·9 to 1·9 for males and from 2·8 to 1·0 for females, a consistent change even more remarkable than for cigarettes in Table III. The patterns are so similar that it is difficult to conceive of any explanation for the inverse behaviour of the intestine and stomach rates than the one already suggested, namely a limited proportion of people at a given age who are susceptible to gastro-intestinal cancer, about the same in all countries, and a preference by certain carcinogens including those in cigarettes and coal to promote malignancy in the intestine excluding the rectum, leading to more intestinal cancers and consequently fewer gastric cancers in the countries where prevalence of those carcinogens in the environment is high. Differences in ratios of gastric to duodenal ulcers, even if they followed a similar pattern, could not explain this since cancer occurs only rarely in the duodenum. Table II shows that the positive correlations between intestinal cancer rates and coal consumption were highly significant in both sexes ($P < 0{\cdot}001$).

When the same analytical procedure is applied to *tea* and *coffee* consumption in pounds per head of the population annually, Table V compares the associations with rates for the intestine and stomach in each sex. A contrast is evident in Table II, the intestine showing strong positive correlation for tea ($P = 0{\cdot}006$ for males and 0·0001 for females), but no significant associations for coffee. Data of consumption were not available for Belgium or South Africa for either commodity, nor for Japan for tea, but Israel has been included giving 18 countries for tea and 19 for coffee.

TABLE V.—*Cancer of Stomach and of Intestine Except Rectum, Age-adjusted Death Rates in Countries Grouped According to Their Annual Consumption of Tea and Coffee*

| Tea or coffee (lb/head) | No. of countries | Male rates | | Female rates | | Total death rates | | Ratio of stomach to intestine | |
|---|---|---|---|---|---|---|---|---|---|
| | | Intestine | Stomach | Intestine | Stomach | M | F | M | F |
| Tea | | | | | | | | | |
| 0– | 7 | 85 | 230 | 82 | 184 | 315 | 266 | 2·7 | 2·2 |
| 0·5– | 3 | 129 | 194 | 117 | 111 | 323 | 228 | 1·5 | 0·9 |
| 1·0– | 4 | 107 | 196 | 114 | 116 | 303 | 270 | 1·8 | 1·0 |
| 2·5+ | 4 | 126 | 190 | 131 | 110 | 325 | 241 | 1·6 | 0·8 |
| Coffee | | | | | | | | | |
| 0– | 6 | 103 | 302 | 105 | 165 | 405 | 270 | 2·9 | 1·6 |
| 2– | 4 | 97 | 279 | 100 | 155 | 376 | 255 | 2·8 | 1·0 |
| 4– | 5 | 114 | 248 | 106 | 133 | 362 | 239 | 2·2 | 1·3 |
| 8+ | 4 | 92 | 273 | 92 | 151 | 365 | 243 | 3·0 | 1·6 |

For *tea* the total gastro-intestinal rates show no appreciable variation but the intestinal rates are low for each sex in the countries with annual consumption below half a pound per head of population and high for each sex where the consumption exceeds 2½ pounds per head. This suggests that a carcinogenic substance more likely to affect the intestine than the stomach is operative in connection with tea drinking as for cigarette smoking and the pollution of food by impurities derived from coal. The stomach/intestine ratios are notably greater in the low consumption areas than in the other areas for each sex. For *coffee* there is no indication of such an effect, neither the gastro-intestinal rates nor the stomach/intestine ratios showing any association with consumption.

*Rectum* (154)

Positive associations occur in each sex with solid fuel consumption ($P = 0{\cdot}05$ for females and 0·08, below confidence level, for males as shown in Table II), these being not as strong as for the rest of the intestine. A carcinogen from coal polluting ingested food appears to affect the intestine as a whole. With the other commodities the relations are weakly negative and insignificant. The three countries with notably high coal consumption, Belgium, United Kingdom and Germany all had high rates for cancer of the rectum (92, 92, 82 for males and 56, 55, 51 for females), but Denmark with the highest rates of all (115, 68) had an average solid fuel level (Table I).

*Pancreas* (157)

There is no significant relation in females with any of the commodities but in males there is a strong positive association with coffee ($P = 0{\cdot}008$). The Scandinavian countries Sweden, Denmark, Norway and Finland with highest coffee consumption (Table I) had male rates for pancreatic cancer of 80, 80, 72, 71 respectively, all above the average, and the female rates in Sweden, Denmark and Finland (55, 54, 52) ranked highest of all countries though for that sex the positive relation with consumption was not significant. Some connection with coffee is suggested by the figures.

*Liver and biliary passages* (155–156)

No positive associations appear for any of the commodities but there are negative relations with cigarettes, significant for males ($P = 0{\cdot}05$) but not for females. In both sexes low death rates occur in Britain and the Commonwealth countries together with high levels of tea consumption and this accounts for the slight negative relation with tea in females ($P = 0{\cdot}08$).

*Larynx* (161)

The only significant associations in Table II are a positive one between female death rates and tea ($P = 0{\cdot}05$), and a negative one with coffee for the same sex ($P = 0{\cdot}005$). As was the case for the mouth and pharynx males show no appreciable relations with these commodities.

*Lung and bronchus* (162–163)

As was to be expected, consumption of cigarettes and of solid fuel are positively associated with lung cancer rates in males. In a previous study of these commodities considered together in relation with male rates in 1958–59 in 19 countries it was found that each factor was correlated independently with the death rates at various ages. At 35–44 for example multiple coefficients as high as 0·8 resulted by combining the coefficients with smoking and air pollution in a ratio of 2 to 1 (Stocks, 1966). From another study of male rates in 20 countries in 1962–63 correlations of 0·38 and 0·70 at ages 55–64 were found with cigarettes and solid fuel respectively, and a partial coefficient of 0·45 with solid fuel when cigarette consumption was held constant (Stocks, 1967). It appears also that effects of smoking a given number of cigarettes tend to be greater in cities such as Liverpool where pollution by coal smoke is high (Stocks and Campbell, 1955).

Table II shows that positive associations also exist between female death rates and solid fuel ($P = 0{\cdot}047$) and, though not significantly, between those rates and cigarettes ($P = 0{\cdot}085$). These have not been studied hitherto for different countries. It is not known however how far these correlations might be affected by differing proportions of women smokers and non-smokers in the various countries, since only the average consumption per adult person can be ascertained from excise data.

Tea shows a positive relation with lung cancer in females ($P = 0{\cdot}02$) and coffee has a corresponding negative relation.

*Bladder* (188)

Several studies comparing mortality from bladder cancer in male smokers and non-smokers in Denmark and U.S.A. have produced indications of a relation with cigarettes (Clemmesen, 1965). Denmark had the highest death rates in 1964–65 in any of the European countries included in the 20 but the consumption of cigarettes there was below the median in 1951–54 (1182). No positive relations between death rates in 1964–65 and consumption of cigarettes 12 years before appear in the 20 countries (Table II).

Solid fuel consumption is strongly correlated however with the death rates in each sex ($P < 0{\cdot}0001$). The six countries with highest mortality were, for males, South Africa, Denmark, United Kingdom, Netherlands, Israel, Belgium (83, 73, 72, 69, 59, 59 per million) and for females Denmark, United Kingdom, Israel, Canada, Netherlands, Belgium (24, 20, 19, 18, 18, 18). Of these Israel had no available data of consumption level but of the other 5 countries in each series all had high levels of coal consumption, though the tobacco level was low in South Africa and Belgium. It is possible that a combined action of carcinogens in tobacco and coal smoke exists as for lung cancer. Tea and coffee consumption show no significant relations with bladder cancer rates.

*Prostate* (185)

Rates for cancer of the prostate are positively associated with the consumption of coffee ($P < 0{\cdot}001$). The country with highest rate is South Africa (186) which was also first in the ranking for bladder in males. The next four are Sweden, Norway, Switzerland and Denmark (178, 165, 158, 156), all with large coffee consumption, followed by Belgium, Australia, France, Netherlands and U.S.A. with moderate levels. Cigarettes show a negative relation with prostatic cancer.

*Ovary* (183·0)

As for prostate there is a strong positive association with coffee ($P = 0{\cdot}006$), and there is also an insignificant relation with solid fuel (0·086) but no positive relations with cigarettes or tea. The 9 countries with highest rates are Denmark,

Sweden, United Kingdom, Netherlands, Switzerland, New Zealand, Canada, U.S.A. and Norway (110, 87, 81, 79, 77, 77, 74, 72, 70) with high coffee consumption in all except 2.

*Breast* (174)

The 10 countries with highest rates for cancer of the breast in females were (in order of ranking and ranging from 256 to 211), Netherlands, United Kingdom, Denmark, Canada, New Zealand, South Africa, Switzerland, U.S.A., Ireland, Belgium, and Table I shows that consumption of cigarettes and solid fuel was above the median for 7 of these and of tea in 6. Strong positive associations with the death rate appeared for solid fuel ($P = 0{\cdot}007$) and tea ($P = 0{\cdot}009$), and an insignificant positive relation with coffee.

*Uterus* (182)

In contrast with the breast there are strong negative associations with cigarette and tea consumption and no relations with solid fuel or coffee. The highest death rates occur in Austria, Denmark, Japan and Italy (177, 176, 135, 130) and the lowest in Norway, Australia, Ireland and Israel (91, 84, 77, 62).

*Leukaemia* (204)

Leukaemia death rates are unrelated with consumption of cigarettes, but have a positive association with solid fuel in males ($P = 0{\cdot}031$) and a negative relation with tea in females ($P = 0{\cdot}05$). Coffee is positively associated with leukaemia in males ($P = 0{\cdot}001$) and in females ($P = 0{\cdot}03$). The first 9 countries ranked in order of their death rates comprise the Scandinavian countries, Israel, Netherlands, U.S.A. and Canada for both sexes, with South Africa for males and New Zealand for females, and of these 7 had high levels of coffee consumption. The 5 with lowest rates in females were Japan, Portugal, United Kingdom, Ireland and Austria in all of which the coffee levels were low.

### REFERENCES

Burrows, T. W.—(1969) *Br. J. Cancer*, **23**, 751.
Clemmesen, J.—(1965) ' Statistical Studies in Malignant Neoplasms '. Copenhagen (Munksgaard).
Gedda, L. and Brenci, G.—(1969) *Acta Genet. med. Gemell.*, **18**, 329.
Segi, M., Kurihara, M. and Matsuyama, T.—(1969) ' Cancer Mortality for Selected Sites in 24 Countries ', No. 5. Department of Public Health, Tohoku University, Sendai, Japan.
Stocks, P.—(1966) *Br. J. Cancer*, **20**, 595.—(1967) *Br. J. prev. soc. Med.*, **21**, 181.—(1968) *Br. J. prev. soc. Med.*, **22**, 406.
Stocks, P. and Campbell, J. M.—(1955) *Br. med. J.*, ii, 923.
Todd, C. F.—(1963) Research paper No. 6. Tobacco Research Council. London.
United Nations Organization—(1957) ' World Energy Supplies ', U.N. Statistical Papers, Series J., No. 2. New York.—(1960) ' World Energy Supplies ', U.N. Statistical Papers, Series J., No. 3. New York.—(1967) ' Commodity Trade Statistics ', Statistical Papers, Series D, Vol. 15 (1965), Vol. 16 (1966). New York.